# Development and characterization of a line with substitution of chromosome 4B of wheat Triticum aestivum L. on chromosome 4Hmar of wild barley Hordeum marinum ssp. gussoneanum (4x)

**DOI:** 10.18699/VJGB-23-66

**Published:** 2023-10

**Authors:** L.A. Pershina, N.V. Trubacheeva, V.K. Shumny, E.D. Badaeva

**Affiliations:** Institute of Cytology and Genetics of the Siberian Branch of the Russian Academy of Sciences, Novosibirsk, Russia Kurchatov Genomics Center of ICG SB RAS, Novosibirsk, Russia; Institute of Cytology and Genetics of the Siberian Branch of the Russian Academy of Sciences, Novosibirsk, Russia Kurchatov Genomics Center of ICG SB RAS, Novosibirsk, Russia; Institute of Cytology and Genetics of the Siberian Branch of the Russian Academy of Sciences, Novosibirsk, Russia; Vavilov Institute of General Genetics of the Russian Academy of Sciences, Moscow, Russia

**Keywords:** Hordeum marinum ssp. gussoneanum, bread wheat, wheat-barley substitution line 4Hmar(4B), Hordeum marinum ssp. gussoneanum, мягкая пшеница, пшенично-ячменная замещенная линия 4Hmar(4B)

## Abstract

Introgressive hybridization is the main method of broadening the genetic diversity of bread wheat. Wild barley Hordeum marinum ssp. gussoneanum Hudson (2n = 4x = 28) has useful agronomical traits, such as high resistance to stress factors, that could be a potential source of new genes for bread wheat improvement. This study aimed to evaluate the possibility of introgression of H. marinum chromosomes into the genome of bread wheat using an incomplete amphiploid H. marinum ssp. gussoneanum (4x)–T. aestivum (Pyrotrix 28) (2n = 54) carrying the cytoplasm of wild barley. For this purpose, we crossed the line of bread wheat variety Pyrotrix 28 with an incomplete amphiploid, and then selected cytogenetically stable 42-chromosome plants with a high level of fertility in hybrid progeny. Genomic in situ hybridization (GISH) revealed a pair of H. marinum chromosomes in the genome of these plants. C- banding analysis confirmed that bread wheat chromosome 4B was replaced by wild barley chromosome 4Hmar. SSR markers Xgwm368 and Xgwm6 confirmed the absence of chromosome 4B, and EST markers BAWU808 and BAW112 identified chromosome 4Hmar in the genome of the isolated disomic wheat-barley substitution line. The study of this line showed that the substitution of chromosome 4B with chromosome 4Hmar resulted in a change of some morphological traits. It included intense anthocyanin coleoptile coloration, specific for H. marinum, as well as a lack of purple coloration of the ears in the leaf sheath, specific for Pyrotrix 28. Line 4Hmar(4B) showed increased performance for several traits, including plant height, number of spikes and tillers per plant, spikelet and grain number in the main spike, grain number per plant, but it had decreased values of 1000-grain weight compared to wheat. Cytogenetic stability and fertility of line 4Hmar(4B) indicated a high compensation ability of barley 4Hmar for wheat chromosome 4B and confirmed their homeology.

## Abstract (ru)

Интрогрессивная гибридизация является основным методом расширения генетического разнообразия мягкой пшеницы. В качестве источника новых генов для мягкой пшеницы может служить дикий ячмень Hordeum marinum ssp. gussoneanum Hudson (2n = 4x = 28), который характеризуется высокой устойчивостью к стрессовым факторам. Настоящая работа посвящена изучению возможности использования неполного амфиплоида H. marinum ssp. gussoneanum (4x)–T. aestivum (Пиротрикс 28) (2n = 54), носителя цитоплазмы дикого ячменя, в качестве источника хромосом H. marinum для их интрогрессии в геном мягкой пшеницы. С этой целью получены гибриды между линией сорта мягкой пшеницы Пиротрикс 28 (далее П28) и неполным амфиплоидом, а затем среди потомков гибридов проведен отбор цитогенетически стабильных 42-хромосомных растений с высоким уровнем фертильности. С использованием GISH-анализа обнаружено наличие пары хромосом H. marinum в геноме этих растений. По результатам С-окрашивания хромосом установлено, что у этой гибридной линии произошло замещение хромосомы 4B мягкой пшеницы на хромосому дикого ячменя 4Hmar. С помощью хромосомспецифичных SSR-маркеров Xgwm368 и Xgwm6 подтверждено отсутствие хромосомы 4B мягкой пшеницы, а с применением EST-маркеров BAWU808 и BAW112 – наличие хромосомы 4Hmar в геноме выделенной дисомной пшенично-ячменной линии. Изучение этой линии показало, что замещение хромосомы 4B мягкой пшеницы на хромосому дикого ячменя 4Hmar привело к изменению ряда признаков: сильно выраженной антоциановой окраске колеоптиле, характерной для дикого ячменя H. marinum, а также отсутствию пурпурной окраски ушек у основания листьев, которая проявляется у линии сорта пшеницы П28. По высоте растений, числу стеблей и колосьев в растении, числу колосков и зерен в главном колосе, а также числу зерен в растении линия 4Hmar(4B) превосходит родительскую линию П28, а по массе 1000 зерен ей уступает. Цитогенетическая стабильность и фертильность линии 4Hmar(4B) указывают на высокую компенсационную способность хромосомы 4Hmar ячменя по отношению к хромосоме 4B мягкой пшеницы и гомеологию между этими хромосомами.

## Introduction

Introgressive hybridization is the main method of broadening the genetic diversity of bread wheat. Identification of new genetic resources for agronomically important traits, such as biotic and abiotic stress resistance, bread-making quality, increased content of microelements in grain and others is an important task (Molnár-Láng, Linc, 2015; Hao et al., 2020). The species of the genus Hordeum L., including annual and perennial barley grasses, as well as grain-type species, are considered a potential source to introgress genes for these traits (Garthwaite et al., 2005; Rubiales, Moral, 2011).

The progamous and embryonic incompatibility is strongly displayed in wheat × barley hybridizations and intergeneric hybrids were produced after the technique to overcome these difficulties had been developed (Kruse, 1973). To date, substitution, addition and translocation lines were developed by hybridization between bread wheat and H. vulgare (Molnár- Láng et al., 2014), H. spontaneum (Taketa, Takeda, 2001), H. chilense (Rey et al., 2015), H. californicum (Fang et al., 2014). Alloplasmic lines (H. vulgare)-T. aestivum were developed and used in breeding to create promising forms and cultivars of wheat (Pershina et al., 2018). In addition, a new grain crop, tritordeum, was produced from hybridization between durum wheat (Triticum durum) and a wild barley, H. chilense (Martín et al., 1999; Martín, 2017).

The ability to cross with wheat is also shown by two subspecies of the sea barley complex H. marinum: herbaceous annuals H. marinum Hudson ssp. marinum (2x) and H. marinum Hudson ssp. gussoneanum (Parl.) Thell. (2x, 4x) (Pershina et al., 2004; Islam et al., 2007). Due to their high resistance to salinity and waterlogging, these species are able to grow in saline meadows and marshes along sea coasts (Garthwaite et al., 2005; Islam et al., 2007). At the same time, salinity tolerance in H. marinum was estimated to be higher than in other species of Triticeae (Garthwaite et al., 2005). In addition, H. marinum ssp. gussoneanum (=H. geniculatum All.) (2n = 28) is resistant to drought and sudden temperature changes (Kobylyanskiy, 1967), and samples with a high protein content in seeds were also isolated (Pershina et al., 2009).

Amphiploids carrying wild barley cytoplasm were produced from hybrids between H. marinum ssp. gussoneanum (4x) and T. aestivum (Pershina et al., 2004) and between H. marinum (2x) and T. aestivum (Islam et al., 2007). The salt- and waterlogging tolerances of amphiploids were intermediate to those of their parents (Islam et al., 2007; Malik et al, 2009) and reduced grain yield was observed due to the negative influence of the H. marinum cytoplasm. To eliminate this cytoplasm effect, it is necessary to produce euplasmic hybrid genotypes, in which wheat is the maternal parent. However, the use of H. marinum ssp. gussoneanum as a pollinator when crossing with wheat is extremely difficult due to the limited amount of pollen in the small flowers of H. marinum.

This paper presents the results of using the incomplete amphiploid H. marinum ssp. gussoneanum–T. aestivum (2n = 54) with wild barley cytoplasm as a source of H. marinum chromosomes for their introgression into the bread wheat genome. The euplasmic wheat-barley disomic substitution line 4Hmar(4B) isolated among the progenies of these hybrids was also studied.

## Materials and methods

Plant material. The line formed from one plant of the wheat variety Pyrotrix 28 (maternal parent, P28) was crossed with individual plants of an incomplete amphiploid L-503 H. marinum ssp. gussoneanum–T. aestivum (P28) (2n = 54). The incomplete amphiploid isolated from the progeny of a cytogenetically unstable amphiploid H. marinum ssp. gussoneanum–T. aestivum (2n = 68–70) carried 42 wheat chromosomes and 12 H. marinum chromosomes, with the exception of 5Hmar (Trubacheeva et al., 2019).

Plants were grown from seeds set in two hybrid combinations (P28 × 503 plant (p)5) and (P28 × 503 plant (p)10), and their progenies were studied in F2–F6 generations. Each generation was formed from the seeds of the most productive plant of the previous generation. Plants were characterized by seed set in the main spike, dividing them according to the level of fertility into groups: CS – completely sterile (no seeds); PS – partially sterile (1–9 seeds); PF – partially fertile (10–19 seeds); F – fertile (20–30 seeds); FF – fully fertile (more than 30 seeds). Starting from F2, the number of chromosomes was counted in plants. The chromosome composition in metaphase I (MI) was determined in euploid (2n = 42) hybrid plants F5 (P28 × 503p10). Cytogenetically stable euploid plants were studied to identify H. marinum chromosomes. Hybridization and the study of the hybrid progeny were carried out in a hydroponic greenhouse.

The isolated wheat-barley substitution line 4Hmar(4B) was studied in the field. The plants were grown on plots 1 m wide, 20 plants per row, with a distance of 25 cm between rows. The control was the parental line P28. Each genotype was grown in three rows arranged in a randomized order. The lines were evaluated according to morphological traits. During harvesting, plant height, tiller number, number of productive spikes, main spike length, spikelet number per main spike, grain number per main spike and per plant were estimated. Statistical analyses were conducted in Microsoft Excel 2007. Single-factor analysis and the calculation of the least significant difference were used (Dospekhov, 1985).

Cytological analysis, genomic in situ hybridization and C-banding. Analysis of chromosome number was performed according to the standard Felgen preparation method using root tips of plants grown in a hydroponic greenhouse. The MI chromosome configuration was examined in pollen mother cells (PMCs) using the 2 % acetocarmine smear method. GISH was performed according to previously published protocols (Trubacheeva et al., 2019). Total genomic H. marinum ssp. gussoneanum DNA was labelled by nick translation with biotin-16-dUTP and used as a probe. The signals were observed and captured using AxioImagerM1 (Zeiss, Germany) fluorescence microscope. The work was performed at the Collective Center for Microscopic Analysis of Biological Objects (ICG SB RAS, Novosibirsk, Russia). C-banding was performed according to a previously published protocol (Badaeva et al., 1994).

DNA marker analysis. Two expressed sequence tag (ESTPCR) markers, BAWU808 and BAW112 (Trubacheeva et al., 2019), were used to identify chromosome 4Hmar of wild barley in the genome of the isolated line. The absence of wheat chromosome 4B in the substitution line 4Hmar(4B) was confirmed using chromosome-specific SSR markers Xgwm368 and Xgwm6 (Röder et al., 1998). The primer sequences were reported in (Trubacheeva et al., 2019. Amplifications were carried out using a Bio-Rad T-100 Thermal Cycler PCR. Amplification products were resolved in 2 % w/v agarose gels and visualized with ethidium bromide. Gel images were captured using a gel documentation system Gel Doc XR+ (Bio-Rad, USA).

## Results

Identification of euploid plants among the progeny of hybrids (P28 × L-503)

As a result of crossing the line P28 with incomplete amphiploid L-503 in four hybrid combinations, shriveled seeds set with a frequency of 3.1 to 6.6 %, from which F1 plants were grown (Table 1).

**Table 1.**
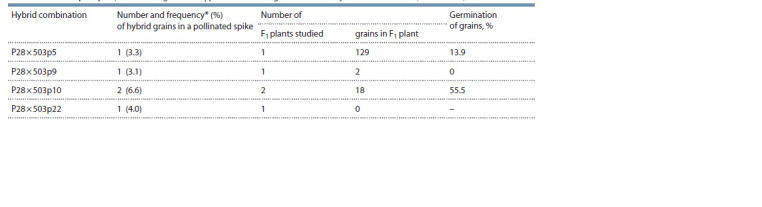
Seed set per spike, number of grains in F1 plants and their germination in hybrid combination (P28 × L-503) From the number of pollinated flowers.

The shriveled seeds set in F1 plants germinated in the hybrid (P28 × 503p5) with a frequency of 13.9 %, and in the hybrid (P28 × 503p10) with a frequency of 55.5 %. Starting from the second generation, the fertility level was studied in the progeny of these hybrids, estimated by the number of seed set per main spike, and the number of chromosomes was analyzed (Table 2).

**Table 2.**
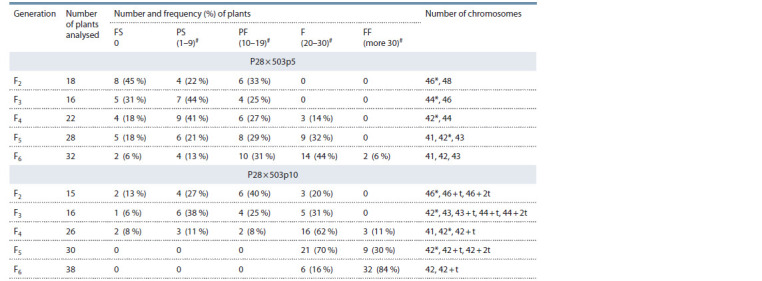
Fertility level and number of chromosomes in F1–F6 of the hybrid combination T. aestivum (P28) (2n = 42) × (H. marinum ssp. gussoneanum × P28) (2n = 54) Number of grains in the main spike; * the most productive cytotypes used to form the next generation.

In the second generation of the hybrid combination (P28 × 503p5), partially fertile (33 %) plants (2n = 46), as well as sterile (45 %) and partially sterile (22 %) plants were observed, among which 46- and 48-chromosome plants were identified. The third generation, derived from a partially fertile plant (2n = 46), also consisted of sterile (31 %), partially sterile (44 %) and partially fertile (25 %) plants. The most productive in F3 was a 44-chromosome plant, among the progeny of which along with sterile (18 %), partially sterile (41 %) and partially fertile (27 %), fertile (14 %) plants were also identified in F4. Among the progeny of the fourth generation, 42- and 44-chromosome plants were found. In F5 and F6 generations derived from fertile 42-chromosome plants, in addition to euploids (2n = 42), aneuploids (2n = 41; 2n = 43) were also identified. At the same time, segregation into plants with different levels of fertility, including sterile ones, was still observed (see Table 2). Fully fertile plants were obtained only in F6 (6 %).

When selecting for productivity, the hybrid combination (P28 × 503p10) had a more accelerated formation of 42-chromosomal cytotypes with a high level of fertility. Fertile 46-chromosome plants (20 %) were already identified in F2 along with sterile (13 %), partially sterile (27 %) and partially fertile (49 %) plants. The remaining groups of plants were represented by cytotypes with chromosome numbers 2n = 46, 46 + t, 46 + 2t.The progenies of the 46-chromosome plant in F3 were sterile (6 %), partially sterile (38 %), partially fertile (25 %), and 42-chromosome fertile (31 %) plants. In F3, in addition to euploids, aneuploids were found, including those with telocentric chromosomes ((2n = 43, 43 + t, 44 + t, 44 + 2t).

Among 26 F4 plants derived from a 42-chromosome plant of the third generation, fertile plants prevailed (62 %), including plants with full fertility (11 %). In total, in F4 of (P28 × 503p10), completely sterile (8 %) plants and plants with a low level of fertility (partially sterile – 11 % and partially fertile – 8 %) only accounted for less than one third. The remaining plants were fertile (62 %) and fully fertile (11 %). In F4 cytotypes with 2n = 41, 42, 42 + t were identified among the studied plants. As a result of the selection of the most productive 42-chromosome plants, the fifth and sixth generations were represented only by fertile plants (in F5 – 70 %, and in F6 – 16 %) and fully fertile plants (in F5 – 30 %, and in F6 – 84 %) (see Table 2).

In order to select meiotically stable 42-chromosome plants for further work, three lines derived from individual plants of the P28 × 503p10 hybrid from F6 were characterized by chromosome configuration at the first meiotic metaphase (MI). Study of meiosis in pollen mother cells (PMC) has shown a high cytological stability of euploid lines. All the studied plants of the line (P28 × 503p10) F6 p1 and the major part of the plants of the line (P28 × 503p10) F6 p2 (79 %) and the line (P28 × 503p10) F6 p3 (89 %) had 21″ bivalents (Table 3). The identified violations are associated with the presence of univalents (20″ + 2′), including those involving telocentric chromosomes (21″ + 2t′).

**Table 3.**
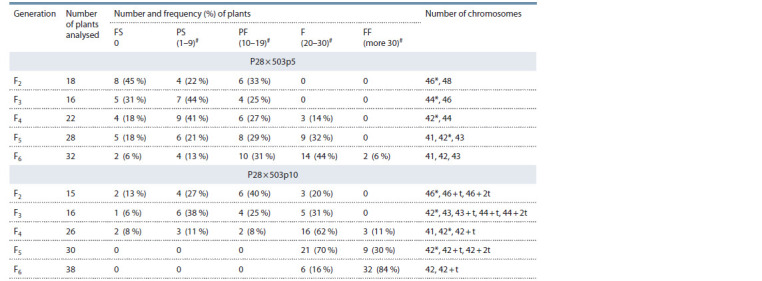
Characteristics of meiosis in lines derived from the hybrid combination (P28 × 503р10) F6

Identification of the wheat–barley disomic substitution line 4Hmar(4B)

**Fig. 1.**
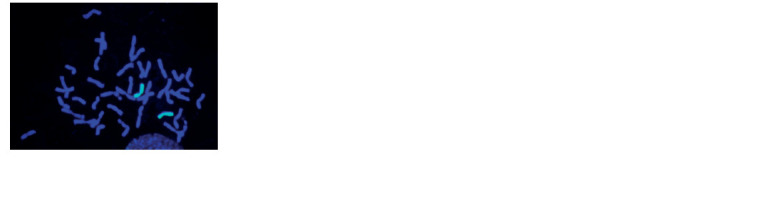
GISH image of a wheat–barley substitution line. Barley chromosomes are in green, wheat chromosomes are in blue.

At the next stage, plants with a 21″ chromosome configuration were included in the work. Using GISH analysis, it was shown that they carry a pair of H. marinum chromosomes (Fig. 1).

**Fig. 2.**
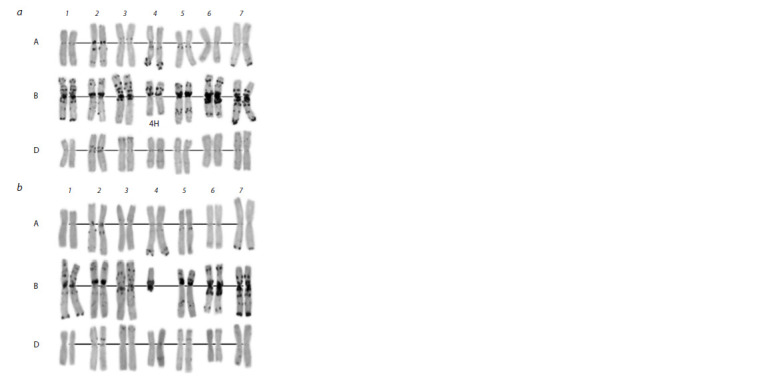
С-banding of the 4Hmar(4B) (2n = 42) (a) and 4Hmar L (2n = 40 + t) (b) substitution lines.

This result indicates that during self-pollination of the progeny of the hybrid (P28 × 503p10) and the production of euploids, a pair of H. marinum chromosomes introgressed into the genome of bread wheat. C-banding identified a pair of chromosomes 4Hmar and the absence of a pair of chromosomes 4B in the isolated line indicating that it was disomic for the substitution 4Hmar (4B) (Fig. 2, a). A telocentric chromosome for the arm of chromosome 4H was identified in a line with 2n = 40 + t chromosomes (see Fig. 2, b).

**Fig. 3.**
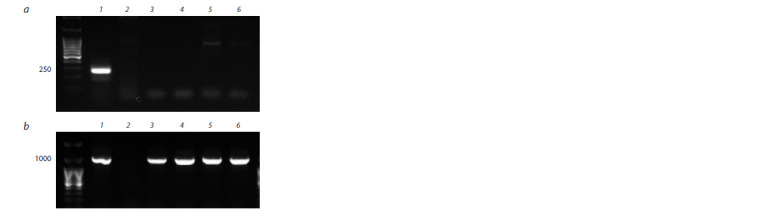
PCR amplification of SSR marker Xgwm368 (a) and EST marker BAWU808 (b). а: 1 – T. aestivum; 2 – H. marinum; b: 1 – H. marinum; 2 – T. aestivum; 3–6 – plants of line 4Нmar(4В). The numbers indicate the fragment length in bp.

Molecular analysis confirmed the presence of chromosome 4Hmar and the absence of chromosome 4B in the genome of disomic wheat-barley lines isolated from self-pollinated progeny of the hybrid combination P28 × 503p10. Molecular markers were used to confirm both the absence of chromosome 4B (Fig. 3, a) and the presence of chromosome 4Hmar (see Fig. 3, b) in isolated substitution lines. The DNA of the parent line P28 and H. marinum was used as a control.

Characterization of the disomic wheat-barley substitution line 4Hmar(4B)

**Fig. 4.**
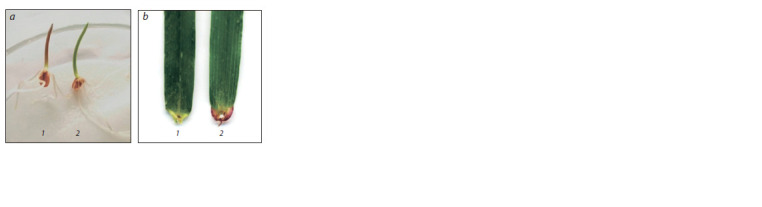
Coleoptile (a); ears at the base of the leaf (b). 1 – line 4Hmar(4B); 2 – line P28.

Plants of the line 4Hmar(4B) differed phenotypically from the parental line P28. Such traits included pronounced anthocyanin coloration of the coleoptile, which is typical for the wild barley H. marinum (Fig. 4, a), as well as an absence of purple coloration of the ears at the base of the leaves (see Fig. 4, b).

**Fig. 5.**
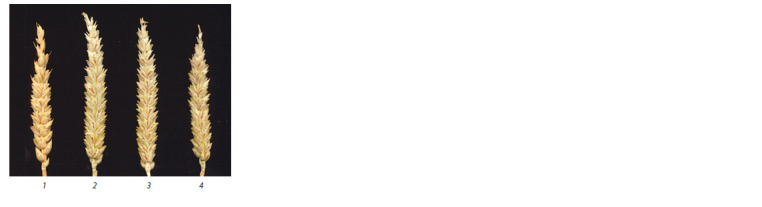
Spikes of lines: 1 – P28; 2–4 – 4Hmar(4B).

**Fig. 6.**
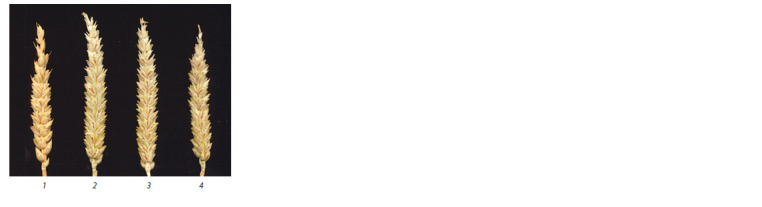
Grains of line P28 (1); line 4Hmar(4B) (2).

Substitution of wheat chromosome 4B for wild barley chromosome 4Hmar resulted in the development of viable plants, in which the values of some quantitative traits were higher than those of the parent line P28. The plant height and yield-related traits in the line 4Hmar(4B) were shown to be significantly different from those of the parental line (Table 4). Thus, the values of plant height, tiller and spike number per plant, spike length, spikelet number per spike, grain number per spike and per plant of the line 4Hmar(4B) were significantly higher than those of P28, while its thousand-kernel weight was less than that in the P28. Figures 5 and 6 show spikes and grains, respectively.

**Table 4.**
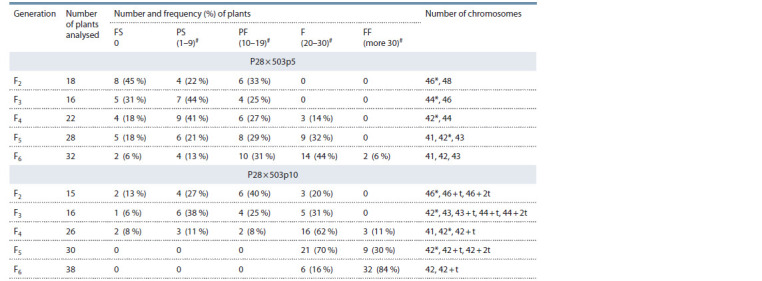
Agronomic characteristics of the wheat–barley substitution line 4Hmar(4B) * р < 0.05.

## Discussion

Wild relatives of bread wheat and their wheat synthetic derivatives are a valuable resource for introgressive hybridization (Davoyan et al., 2012; Molnár-Láng, Linc, 2015; Li et al., 2018). Tritordium is used in hybridization with wheat to transfer H. chilense chromosomes into the wheat genome (Martin, 2017), and triticale is used to transfer rye chromosomes (Shchapova, Kravtsova, 1990).

Earlier in our work, amphiploid H. marinum ssp. gussoneanum– T. aestivum (2n = 70) wild barley cytoplasm was used as a maternal parent in crosses with bread wheat to obtain alloplasmic disomic wheat–barley substitution lines 7Hmar(7B), 7Hmar(7D), 7HmarL(7D), as well as ditelosomic addition lines 2n = 42 + 2t (7HmarL) (Pershina et al., 2004; Trubacheeva et al., 2019). In this work, individual plants of the incomplete amphiploid H. marinum ssp. gussoneanum– T. aestivum (2n = 54) with wild barley cytoplasm were used as pollinators when crossing with the line P28 to introduce the genetic material of H. marinum into the euplasmic genetic background of bread wheat. The frequency of hybrid seed set was low, but some of the F1 hybrid plants were viable, and two F1 hybrids (P28 × 503p5) and (P28 × 503p10) set seeds that germinated.

The analysis of the obtained data revealed differences in the process of formation and the rate of cytological stabilization between the progeny of hybrids (P28 × 503р5) and (P28 × 503p10). This process was slower in the hybrid combination (P28 × 503p5) compared to the combination (P28 × 503p10). Thus, the progeny of 42-chromosome plants of the hybrid (P28 × 503p5) in the F5 and F6 segregated in plants with different levels of fertility, including completely sterile ones. In F6, only half of 32 plants were fertile and fully fertile. In the hybrid combination (P28 × 503p10), on the contrary, only fertile or fully fertile plants were obtained in the fifth and sixth generations already. In addition, in the progeny of the hybrid (P28 × 503p10), in contrast to (P28 × 503p5), barley telocentric chromosomes appeared. Such results are consistent with the data of the authors who emphasized the uniqueness of the progeny of each hybrid grain as a source of diversity in the development of wide hybrid derivatives (Shchapova, Kravtsova, 1990).

As follows from the results obtained, the process of stabilization of karyotypes of 42-chromosome plants in the progeny of the (P28 × 503p10) F6 hybrid was accompanied by the substitution of a pair of wheat chromosomes for a pair of H. marinum chromosomes. It was shown using GISH, C-banding and molecular analysis that chromosome 4B was substituted by chromosome 4Hmar in cytogenetically stable euploid plants. In addition, it was found that telocentrics also belong to the chromosome 4Hmar.

The type of wheat-barley substitution was also confirmed by the morphological traits that were exhibited in the line 4Hmar(4B). The absence of purple coloration of the ears at the base of the leaves in the line 4Hmar(4B) indicates the absence of wheat chromosome 4B, since this trait is controlled by the Ra2 gene located on this chromosome (Melz, Thiele, 1990) and exhibited in P28. The smaller grain in the substitution line 4Hmar(4B) can be associated both with the absence of chromosome 4B, which affects the grain size and shape in wheat (Rahman et al., 2020), and with the presence of chromosome 4Hmar, because H. marinum belongs to small-seeded bar-ley grasses (Bothmer et al., 1991).

The line 4Hmar(4B) had a clear phenotypic marker specific for H. marinum. This is anthocyanin coleoptile coloration, which is absent in the parent line P28 and was previously found in the alloplasmic wheat–barley substitution line 7Hmar(7D) (Khlestkina et al., 2011). The accumulation of anthocyanin in vegetative organs is positively related to resistance to stress factors, and in wheat, ability to accumulate anthocyanins in the coleoptile is controlled by the Rc (red coleoptile) genes (Khlestkina et al., 2011). In this regard, the line 4Hmar(4B) may be useful for future studies of resistance to abiotic stresses, because H. marinum contains resistance genes (Garthwaite et al., 2005; Islam et al., 2007; Malik et al., 2009).

It has been established that homoeologous group 4 chromosomes of other barley species also possess genes that could be used for wheat breeding. For instance, chromosome 4Hch of H. chilense contains genes for resistance to Septoria tritici and salt stress (Said, Cabrera, 2009), and chromosome 4H of H. vulgare was able to increase water use efficiency associated with drought tolerance of a wheat substitution line (Molnár et al., 2007). The line 4Hmar(4B) obtained in our work may have a potential for breeding, since it is characterized by high yield. Thus, the values of the number of spikes, the length of the spike, the number of spikelets per spike, the number of grains per and per plant in the 4Hmar(4B) line are higher than those in the line P28. These results, as well as the cytogenetic stability of the line 4Hmar(4B), indicated homoeology and high ability of barley 4Hmar chromosome to compensate for wheat 4B.

## Conclusion

Thus, the efficiency of using the incomplete amphiploid (H. marinum ssp. gussoneanum–T. aestivum) (2n = 54) with wild barley cytoplasm to transfer H. marinum chromosomes into euplasmic genetic background of bread wheat has been shown.

## Conflict of interest

The authors declare no conflict of interest.
